# Effectiveness of adjuvant radiotherapy in patients with oropharyngeal and floor of mouth squamous cell carcinoma and concomitant histological verification of singular ipsilateral cervical lymph node metastasis (pN1-state) - A prospective multicenter randomized controlled clinical trial using a comprehensive cohort design

**DOI:** 10.1186/1745-6215-10-118

**Published:** 2009-12-23

**Authors:** Maximilian Moergel, Antje Jahn-Eimermacher, Frank Krummenauer, Torsten E Reichert, Wilfried Wagner, Thomas G Wendt, Jochen A Werner, Bilal Al-Nawas

**Affiliations:** 1 Department of Oral and Maxillofacial Surgery, University of Mainz, Medical Center, Augustusplatz 2, D-55131 Mainz, Germany; 2 Institute of Medical Biostatistics, Epidemiology and Informatics (IMBEI), University of Mainz, Medical Center, Obere Zahlbacherstr. 69, D-55131 Mainz, Germany; 3 Department of Medical Biostatistics and Epidemiology, University of Witten-Herdecke, Alfred-Herrenhaus Str. 50, D-58448 Witten-Herdecke, Germany; 4 Department of Oral and Maxillofacial Surgery, University of Regensburg, Medical Center, Franz Josef Strauß Allee 11, D-93053 Regensburg, Germany; 5 Department of Radiation Oncology, University of Jena, University Hospital, Bachstr. 18, D-07743 Jena, Germany; 6 Department of Otolaryngology, University of Marburg, Medical Center, Deutschstraße 3, D-35037 Marburg, Germany

## Abstract

**Background:**

Modern radiotherapy plays an important role in therapy of advanced head and neck carcinomas. However, no clinical studies have been published addressing the effectiveness of postoperative radiotherapy in patients with small tumor (pT1, pT2) and concomitant ipsilateral metastasis of a single lymph node (pN1), which would provide a basis for a general treatment recommendation.

**Methods/Design:**

The present study is a non-blinded, prospective, multi-center randomized controlled trial (RCT). As the primary clinical endpoint, overall-survival in patients receiving postoperative radiation therapy vs. patients without adjuvant therapy following curative intended surgery is compared. The aim of the study is to enroll 560 adult males and females for 1:1 randomization to one of the two treatment arms (irradiation/no irradiation). Since patients with small tumor (T1/T2) but singular lymph node metastasis are rare and the amount of patients consenting to randomization is not predictable in advance, all patients rejecting randomization will be treated as preferred and enrolled in a prospective observational study (comprehensive cohort design) after giving informed consent. This observational part of the trial will be performed with maximum consistency to the treatment and observation protocol of the RCT. Because the impact of patient preference for a certain treatment option is not calculable, parallel design of RCT and observational study may provide a maximum of evidence and efficacy for evaluation of treatment outcome. Secondary clinical endpoints are as follows: incidence and time to tumor relapse (locoregional relapse, lymph node involvement and distant metastatic spread), Quality of life as reported by EORTC (QLQ-C30 with H&N 35 module), and time from operation to orofacial rehabilitation. All tumors represent a homogeneous clinical state and therefore additional investigation of protein expression levels within resection specimen may serve for establishment of surrogate parameters of patient outcome.

**Conclusion:**

The inherent challenges of a rare clinical condition (pN1) and two substantially different therapy arms would limit the practicality of a classical randomized study. The concept of a Comprehensive Cohort Design combines the preference of a randomized study, with the option of careful data interpretation within an observational study.

**Trial registration:**

ClinicalTrials.gov: NCT00964977

## Background

Ninety percent of all tumors in the head and neck region are of squamous cell origin and world wide annual incidence is estimated at 363.000 with a mortality of 200.000 [1,2]. Oropharyngeal squamous cell carcinoma (OSCC) is the predominant malignancy in the head and neck region, and is the seventh most frequently occurring tumor world-wide. In the Federal Republic of Germany, up to 3-5% of all malignant tumors are found within the oral cavity and the oropharynx, with 7.500 new cases annually. 2.2% cases of death occur as a consequence of oropharyngeal malignancy [3]. Thus, treatment of patients with OSCC is of great medical and economic importance. Prevailing curative therapeutic strategies combine radical resection of the tumor with a safety margin, followed by radiation of the original tumor site and adjacent locoregional lymphatic drainage areas [4-6]. To date, selection of the individual therapeutic pattern is essentially guided by pre- and post-therapeutic TNM staging parameters. Particularly for advanced tumors, postoperative radiotherapy, possibly combined with chemotherapeutic agents, is favorable and recommended [7-9]. Interdisciplinary guidelines state the following detailed recommendations for application of adjuvant radiation therapy:

- non in sano resection if reoperation is impossible (R1-, R2-status)

- primary tumor status > pT2 and pN2, pN3

- extranodular spread of the disease

- lymphangiosis carcinomatosa

- facultative: pN1

According to these recommendations, postoperative radiotherapy of advanced tumors is advised, while for small tumors the indication depends on further parameters such as the pN- state. In these cases, verification of metastasis in more than a single lymph node (pN2) leads to additional radiotherapy. In tumors with a diameter of less than 4 cm (T1, T2) and concomitant verification of a single lymph node metastasis, no explicit therapeutic recommendation is presently offered, leaving radiation as an optional complement for these cases. Meta-Analysis revealed only a few studies taking this special group of patients into account, the results of which suggest that adjuvant radiotherapy is an additional risk factor for overall survival. However, the small patient count, inhomogeneous group distribution and ambiguous risk factors in these studies contribute to a substantial bias. Due to the emerging importance of quality of life information, the overall survival rate is not the only parameter of interest when reviewing the effectiveness of modern tumor therapy. Therefore, there is urgent need for controlled studies verifying the effect of postoperative radiotherapy. The aim of the present study is to investigate the clinical outcome in patients with or without postoperative radiotherapy after curative intended radical surgery, because meta-analysis of present retrospective clinical data failed to support a safe and justifiable treatment regime in patients with small tumor (T1, T2) and a single ipsilateral lymph node metastasis.

The following key methodological problems have to be addressed in the protocol:

• extremely different treatment arms with strong preferences for one or the other therapy arm

• low number of eligible patients per center

• Inclusion criterion (pN1), including a pathological diagnosis and surgical treatment with possible differences

• Radiation treatment as one arm with special quality control needs

The purpose of presenting this paper is to discuss how these specific issues were addressed.

## Methods/Design

### Study Design

The study is designed as a non-blinded, prospective, randomized controlled clinical trial. Study approval was given by the Ethics Committee of the State Medical Council of Rhineland-Palatinate, Germany [Ref. No: 837.148.03 (3810)]. As part of the Johannes Gutenberg-University, the Coordination Center for Clinical Trials (KKS Mainz, Germany), will monitor the study progress and assure data accuracy. Obvious side effects of radiation therapy render blinding of patients and examiner impossible.

### Study objectives

The objective of the clinical study will be to investigate two different patient groups (irradiated/unirradiated) with pT1/2 primary and verification of a singular ipsilateral lymph node metastasis in parallel design in order to evaluate the possible benefit of radiation therapy. Subsequent investigation of biological parameters will be performed to assess the capacity to predict tumor progression and to evaluate surrogate markers of radioresistance. The following null hypothesis forms the basis of the present study: Radiation therapy will have no influence on the overall survival in patients with pT1/2, pN1 primary tumor. Secondary outcome variables include incidence and time to tumor relapse (locoregional relapse, lymph node involvement and metastatic spread), Quality of life as reported by EORTC (QLQ-C30 with H&N 35 module) and time from operation to orofacial rehabilitation.

### Patients

Males and females with a histologically verified diagnosis of a primary squamous cell carcinoma of the oral cavity or the oropharynx are eligible. Inclusion criteria are as follows:

- maximum tumor diameter less than 4 cm in the pathohistological specimen irrespective of histological grading (pT1 or pT2)

- concomitant histological verification of a singular ipsilateral lymph node metastasis less than 3 cm in diameter (pN1) without penetration of the lymph node's capsule and without presence of lymphangiosis carcinomatosa

- radical resection of the tumor within adequate resection margins (R0)

- written informed consent from the patient

- adequate performance status ECOG Index ≤ 2

Patients younger than 18 and pregnant women are to be excluded. Further criteria of exclusion are reported drug addiction or intake of remedies with a potential influence on compliance or impaired judgment. In addition, patients with familial or job related responsibilities which may preclude the patient from maintaining the study schedule, as well as patients with inadequately treated diseases, such as untreated diabetes mellitus or acute heart insufficiency (ECOG-Index>2), will also be excluded.

### Study interventions

Radiotherapy should begin within 6 weeks after the last surgical intervention while respecting a minimal postoperative healing period of at least 8 days. Causes for delay of intervention are to be documented. In both the RCT and observational study, patients of group 1 will be treated by irradiation and patients of group 2 will not receive radiotherapy.

### Surgical treatment

**Figure 1:**
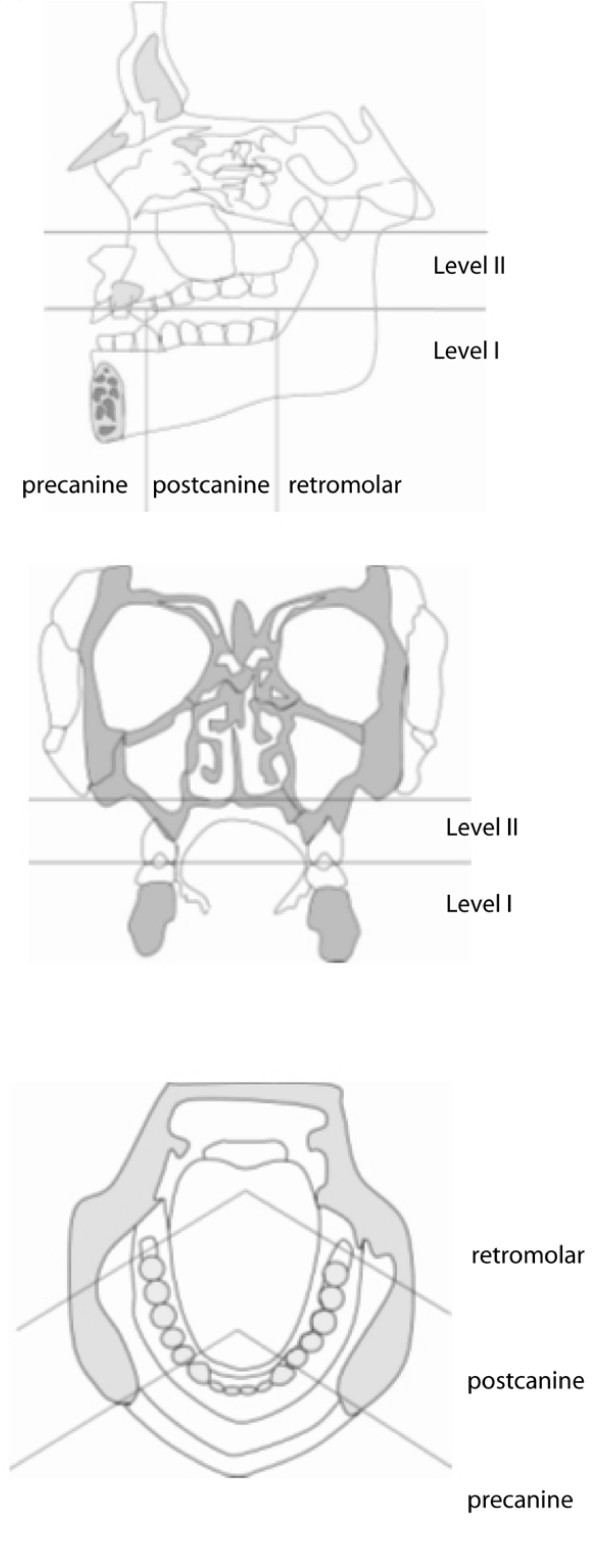
**According to Bier (1982) description of tumor position follows its relation to the occlusal plane, canine and the last molar in transversal, saggital and axial projection**.

**Figure 2:**
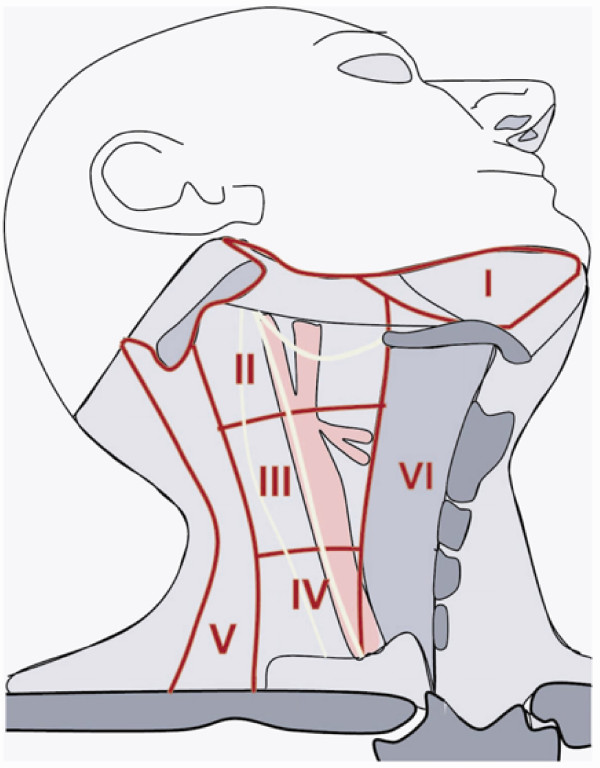
**Definition of the cervival levels as defined by Robbins 1991**.

The primary tumor is considered radically resected if macroscopic and histologic evaluation shows resection margins without tumor residuals (R0 status). For surgical proceeding, a safety margin of at least 0.5 cm is established. Lymph node resection will be oriented in relation to the tumor center, which can be localized in level I (below occlusional plane) or level II (above occlusional plane), in addition to being precanine, postcanine or retromolar (Figure 1). Infiltration of the periosteum and midline crossing is also characterized. Classification of cervical regions follows the recommendations of Robbins from 2002 [10]. Hence, the neck is divided into 6 different sections (Figure 2).

#### Ipsilateral N0 state of cervical lymph nodes

Pre- and post-canine localized tumors of level I require selective neck dissection of the cervical level 1-3. For Level I tumors of the retromolar triangle selective neck dissection of ipsilateral level 1-5 is proceeded. For level II tumors no selective neck dissection is considered.

#### Contralateral N0 state

No neck dissection is performed

#### Ipsilateral N1-N2 state

For retromolar localized level I tumors, a modified radical neck dissection is performed. If the tumor is localized, pre- or post-canine, intraoperative frozen sections of level 1 lymph nodes are evaluated. Positive intraoperative results lead to modified radical resection of level 1-5 lymph nodes, while negative results only require resection of the lymph nodes within level 1-3. Negative intraoperative frozen sections in level II tumors require no lymph node dissection, but all metastatic affection of level II-III lymph nodes lead to modified radical neck dissection.

#### Contralateral N1-N2 state

Frozen sections will be performed for level 1 lymph nodes. Modified radical neck dissection is performed in positive results, resection of level 1-3 lymph nodes is conducted in case of negative results. Incidence of metastasis in level 2-3 lymph nodes acquire modified radical neck dissection.

#### Lymph node therapy of midline tumors

Midline tumors require bilateral resection of lymph node echelons at risk. Bilateral modified neck dissection is performed for staging purposes if indicated. Contralateral lymph node dissection of the level 1-3 in precanine localized tumors is mandatory.

### Technical conditions and practical execution of radiation therapy

Radiotherapy is performed using photons of 4 to10 MeV or/and electrons of 6 to 15 MeV maximum energy. Individual 3-dimensional dose distribution calculations are mandatory and have to be made on the basis of a postoperative native computer tomography. All patients are to be treated while immobilized by a custom made face mask made of thermoplast or equivalent. Clinical target volume definition encompasses the original tumor site with a safety margin of 2 cm in each direction.

### Floor of mouth, anterior tongue

Irradiation of the anterior two third of the tongue as well as the anterior floor of mouth is irradiated by laterally opposing beams, while the maxilla is separated by insertion of a bite block.

### Oropharynx, buccal plane, soft palate confined to one side

For tumors of the buccal plane, the tonsilla, the soft palate and the retromolar triangle, two types of target volume planning may be applied.

An ipsilateral target volume for the primary is defined, including the lymph nodes of group IB, IIA and IIB. The irradiation technique recommended uses two wedge fields, typically angulated by 90 to 120 degrees. The ipsilateral node levels III-V are treated by an anterior portal down to the clavicle, while the contralateral neck is spared.

Tumors of the lateral and dorsal pharyngeal wall and their lymphatic drainage (levels II, III, IV and V) are irradiated bilaterally with opposing beams. Irrespective of technique used, 3-D conformal dose shaping is highly recommended.

### Dosage and fractionation

Target volume definition and dosage must be performed according to the rules of ICRU report 50. At the primary tumor site and at involved lymph node levels, a total dose of 59.4 Gy in 33 fractions within 45 days is scheduled, and for electively irradiated volumes, a total dose of 50.4 Gy in 28 fractions is scheduled. All fractions of 1.8 Gy are given five times per week. In case of machine break down, an additional fraction per week is recommended with a minimum 6 h interval between two fractions on the same day or on Saturday. Total treatment duration (including planned/unplanned interruptions) is to be documented in days. All patients must be available for intent to treat analysis.

Instead of the classical portal arrangements and dose distributions, intensity modulated radiotherapy using an inverse calculation algorithm may be used in experienced centers. Dose constraints for normal tissues are left to the discretion of the participating centers, but dose specification according to the RTOG protocol H 0022 is recommended [11]. These details will be given in a standard operation procedure.

### Follow-Up

The recruitment phase will last 4 years. The follow up will include physical examinations, ECOG index, and quality of life questionnaires collected at 3, 6 and 12 months post operative. Further follow up will be performed annually until the study end. This means that patients will be followed up for a minimum of 5 years and a maximum of 9 years.

### Randomization and Sample Size

The present study design consists of a two-armed, randomized controlled trial (RCT), but patients who refuse to participate in a randomized approach and who express a radiation preference will be included in a prospective observational study after giving informed consent. This parallel observational trial will follow the same treatment and observation protocol as the RCT. Patients who agree to the randomized trial will be randomized and stratified according to lymph node therapy (yes/no) as formulated by the DOESAK [12]. Randomization to the radiation group and control group will be done 1 to 1, with recruitment extending over a period of 4 years and ongoing, annual follow-up continuing until the trial ends, i.e. for at least 5 years for each patient. Sample size calculation will be performed for the two-sided log rank test at a significance level of 5%. Assuming exponential distributed survival with 45% survival within the control group and 55% within the radiation group after 5 years (as per results observed in the DOESAK collective), and a drop-out rate of 5% per year, 280 patients per group are required to detect a difference of 5% in overall survival with a power of 70%. To ensure proper power adjustment, an intermediate analysis of the true drop out rate will be conducted. Recruitment of at least 280 patients per group in the observational study will be pursued only in the unfortunate event that patient recruitment within the randomized trial fails.

### Analysis of RCT and observational trial

Carrying out this RCT is only justifiable if there is a realistic chance of enrolling enough patients to complete a meaningful statistical analysis. Therefore, an intermediate assessment of the randomized patient population will be performed after the first and second years of recruitment. If the numbers are deemed to be insufficient, additional study sites will be added and a web-based information platform will be launched in the hopes of garnering additional attention from affected patients and physicians.

If less than 5% of patients agreed to randomization within the first year and less than 10% within the second year, the RCT will be stopped and continued solely as an observational trial (see Table 1). Due to the dual study design (parallel RCT and observational cohort), the following analysis scenarios are possible:

**Table 1:** Time Schedule

Start	-> Start of recruitment phase (presumably Sep. 2009)
End of year 1	**->**Intermediate assessment of recruitment level:
	- If fraction of randomized patients <5% of total count: ongoing recruitment, else abortion of randomization

End of year 2	**->**Second intermediate assessment of recruitment level:
	- If fraction of randomized patients <10% of total count: ongoing recruitment, else abortion of randomization

End of year 4	**-> End of recruitment phase**

	-

Year 5+6	**->**Follow-up
Year 7+8	
Year 9	
End of year 9	**-> End of Follow-Up**

	**-> Final analysis of the primary clinical endpoint: Evaluation of the overall-survival**

1) If a sufficient number of patients consent to randomization, treatment comparison will be performed within the randomized arms. The non-randomized cohort will be used to evaluate the external validity of observed treatment effects according to Schmoor et. al. [11].

2) If a sufficient number of patients cannot be randomized, all recruited patients will be analyzed. Descriptive comparison of treatment groups will be performed for all baseline variables. All analyses will be performed with adjustment to baseline variables affecting survival. Results will be interpreted with respect to any group differences in baseline variables. This study setup was first described by Schmoor et al. as a comprehensive cohort study design [13].

## Outcome measurements

The primary analysis will be an intention to treat analysis on the randomized subjects.

### Primary clinical endpoint

Overall survival at the end of the study

### Secondary clinical endpoints

#### Incidence of tumor relapse

- Time to occurrence of lymphatic metastases

- Time to occurrence of local relapse

- Time to occurrence of distant metastasis

- Tumor related death due to local tumor progression or metastatic spread

#### Life quality

◦ Time to provisional orofacial rehabilitation

◦ Time from operation to first intermediate prosthesis intake

◦ Time from operation to definitive prosthesis application

◦ Life Quality report (EORTC QLQ-C30 with H&N 35 module)

### Metabolic factors

Since the patients involved have equal tumor states, immunohistological investigation of protein expression levels of paraffin embedded sections may help to establish surrogate parameters of radioresistance in oral squamous cell carcinoma. The following factors are to be considered: ki-67, GLUT-1, Hif-1alpha, MCT-1, p53-family members, RB/p130, adhaesion molecules, cytokine and cytokine-receptor expression (IL-2/IL2R_), eIF4E. Pathohistological evaluation of tumor host interaction will be described by vessel density, amount of infiltration by dendritic cells and macrophages, and chain expression in TIL, Fas/FasL-Expression.

### Statistical analysis

Overall survival will be evaluated using a Cox proportional hazard model with treatment and adequate lymph node therapy (yes/no) as covariates. Further covariates may be considered if they affect survival. Statistical analysis will be done with SAS and SPSS^® ^(Statistical Package for Social Science) software for Windows (Chicago, II USA). The Institute of Medical Biostatistics, Epidemiology and Informatics (IMBEI) at the University Medical Center of the Johannes-Gutenberg-University Mainz will provide advice for statistical analysis.

## Discussion

Randomized (blinded) clinical trials represent the gold standard for comparison of treatment efficacy as postulated by the criteria of modern evidenced based medicine [14]. Therefore, RCTs are desired to establish "Grade A" treatment recommendations. A key advantage of randomization is the equal distribution of known and unknown confounding variables within intervention groups, and the elimination of their influence on the outcome [15]. Only the experimental design of a RCT reduces the otherwise unavoidable risk of selection bias and systematic differences which are not induced by the treatment itself, but may affect the outcome. Thus, the study described in this paper could provide a reliable, unbiased appraisal of radiation effectiveness. However, field experience from studies with strongly differing therapy regimens have shown that randomization might fail due to a strong preference of patient and/or physician for a certain therapeutic option (preference effect) [15-17]. Within the "German Breast Cancer Study Group" (GBSG) which investigated operation vs. radiation, only 6.4% of eligible patients consented to randomization [18,19]. Conducting such a study as RCT might not be suitable as a source of evidence due to essential loss of statistical power. External generalization of such data might be harmful if comparison to the intended target population is not supported by a sufficient patient count. Unfortunately the magnitude of the preference effect can not be estimated in advance, although its occurrence in the present study is highly probable due to similarities to the German Breast Cancer Study Group. Thus, the present study setup also uses a comprehensive cohort design. In the unfortunate event that the study fails as a RCT, the obtained clinical data will still allow careful evaluation of radiation effectiveness under controlled conditions in specialist centers. Furthermore, this valuable clinical data might serve as a useful guide for the design of controlled clinical trials in the future. Using a comprehensive cohort design, Rauschecker et al. enrolled more than 1000 patients within the "German Breast Cancer Study Group" (GBSG), and presented a valuable outcome after a long observational period [19].

Further key methodological aspects included differing therapeutic regimens between the study centers, as varying surgical strategies may result in a performance bias with influence on the outcome [20]. The variability of surgical procedures in the head and neck area for control of loco-regional relapse and lymph node involvement is high [21,22]. Thus balanced randomization with stratification by the surgical treatment recommendations of Bier [12] serves for additional improvement of internal validity. In addition, radiation therapy is a well established subsequent therapeutic option for patients with advanced head and neck cancer, whereas a positive effect for the T_1-2_N_1 _neck is still open to evaluation [7]. Similar to surgery, diverse radiation protocols and additional physical considerations necessitate a singular treatment center to ensure quality control as crucial aspect of quality assurance for the present study.

## Conclusion

Under the constraints of a rare condition (pN1) and two substantially differing therapy arms, the practicality and obtainable evidence of a classical randomized study are challenged. The concept of a "Comprehensive Cohort Design" includes the option of a randomized study with an observational study as a backup if randomization of patients fails due to a strong preference effect.

## Abbreviations

OSCC: oropharyngeal squamous cell carcinoma; GBCS: German Breast Cancer Study Group; RCT: Randomized clinical trial; IZKS: Interdisciplinary Center for Clinical Trials; EORTC: European Organisation for Research and Treatment of Cancer; QLQ-C30: Quality of life Questionaire C30; ECOG Index: Eastern Cooperative Oncology Group Index.

## Competing interests

The authors declare that they have no competing interests.

## Authors' contributions

MM coordinates the study centers and helped to draft the manuscript. AJE participated in the design of the study. FK participated in the design of the study. TER conceived of the study, and participated in its design. WW conceived of the study, and participated in its design.

TGW conceived of the study, and participated in its design. JAW conceived of the study, and participated in its design. BA drafted the study protocol, conceived of the study, and participated in its design. All authors read and approved the final manuscript.
